# Milk of magnesia in enhanced recovery after surgery for preventing postoperative ileus after hysterectomy: randomized controlled trial

**DOI:** 10.1007/s00520-026-11023-z

**Published:** 2026-07-18

**Authors:** Wathirada Karnchanabanyong, Irene Ruengkhachorn, Nida Jareemit, Jiraporn Thurakit, Darisa Sriclung, Raveewan Sangprasert, Natchanon Sathapanapitagkit, Jarin Ketthong, Sompop Kuljarusnont

**Affiliations:** 1 Department of Obstetrics and Gynecology, Faculty of Medicine Siriraj Hospital, Mahidol University, 2 Wanglang Road, Bangkok Noi, Bangkok, Thailand; 2 Division of Obstetrics and Gynecology Nursing, Department of Nursing, Siriraj Hospital, Mahidol University, Bangkok, Thailand; 3 Department of Pharmacy, Siriraj Hospital, Mahidol University, Bangkok, Thailand

**Keywords:** Enhanced recovery after surgery, ERAS, Gynecologic malignancy, Hysterectomy, Milk of magnesia, MOM, Postoperative ileus, POI

## Abstract

**Purpose:**

To evaluate whether adding milk of magnesia (MOM) to an enhanced recovery after surgery (ERAS) pathway prevents postoperative ileus (POI) and improves postoperative bowel function.

**Methods:**

This randomized controlled trial at the Gynecology Unit, Siriraj Hospital (Bangkok, Thailand) enrolled patients undergoing hysterectomy for benign or malignant indications during September 2023 through February 2024. Participants were randomly assigned to ERAS alone (*n* = 80) or ERAS plus MOM (*n* = 80). The MOM group received 15 mL on postoperative day 0 and then 30 mL twice daily until first flatus or bowel movement. The primary outcome was the time from surgery to resumption of a well-tolerated solid diet. Secondary outcomes included POI incidence, time to first flatus, patient-reported outcomes, and MOM-related complications.

**Results:**

Among 160 patients, 60 (37.5%) had gynecologic malignancies: endometrial cancer (*n* = 34), ovarian cancer (*n* = 16), cervical cancer (*n* = 8), and leiomyosarcoma (*n* = 2). Time to well-tolerated solid diet was significantly shorter in the MOM group compared with the control group (20.6 ± 10.9 vs 28.8 ± 16.3 h; *P* < 0.001). The incidence of POI was also significantly lower (3.8% vs 27.5%; *P* < 0.001), and time to first flatus was shorter (17.1 ± 7.9 vs 22.3 ± 10.8 h; *P* < 0.001). Patient-reported outcomes favored the MOM group, with higher eating and daily activity satisfaction scores. No severe adverse events occurred in the MOM group.

**Conclusion:**

Adding MOM to ERAS was associated with reduced POI incidence and faster recovery of bowel function in patients undergoing abdominal hysterectomy for benign or malignant conditions.

**Trial registration:**

Thai Clinical Trials Registry (TCTR: TCTR20230816005), registered on 16 August 2023.

## Introduction

Postoperative ileus (POI) is a frequent complication after abdominal surgery, occurring in 10%–30% overall and reaching 40%–51.8% after gynecologic cancer procedures [1–3]. POI increases postoperative morbidity and prolongs the length of hospital stay, constituting a public health concern [1–3]. Symptoms include nausea, vomiting, bloating, delayed passage of flatus and stool, abdominal distention, and abdominal tenderness [1]. The pathophysiology is multifactorial. Peritoneal irritation, bowel manipulation, intraoperative autonomic stimulation, exposure to opioids and anxiolytics, and postoperative stress reduce bowel smooth-muscle motility, which typically normalizes within 2–3 days [1, 4]. Several nonpharmacologic and pharmacologic strategies, including coffee, juice, tea, gum chewing, prokinetic drugs, ginger, and oral magnesium hydroxide, have been investigated to hasten restoration of bowel function and gastrointestinal motility [2, 5–10]. The enhanced recovery after surgery (ERAS) protocol is now the standard of care for gynecologic surgery for both benign and malignant conditions [11, 12].

Milk of magnesia (MOM) is an oral magnesium hydroxide formulation. Each 5 mL contains 400 mg magnesium hydroxide, 0.25 mg each of methylparaben and propylparaben, 0.0025 mL peppermint oil, and purified water to 5 mL. MOM is a low-cost laxative with minimal adverse effects. Therapeutic uses include laxation at 30–60 mL once daily and acid neutralization at 5–15 mL 4 times daily. MOM has shown efficacy in preventing ileus after benign and malignant gynecologic operations [2, 13]. In a reported case of radical hysterectomy, 30 mL MOM twice daily with bisacodyl suppositories hastened bowel function recovery and permitted discharge without significant complications [13]. However, evidence on incorporating MOM into ERAS pathways remains limited.

The primary objective was to compare time to solid diet resumption between groups. The secondary outcomes were POI incidence, time to recovery of bowel function, postoperative complications, eating satisfaction score, daily activities satisfaction score, length of hospital stay, and MOM-related complications.

## Methods

### Study design and oversight

After authorization from the Siriraj Institutional Review Board (Si-555/2023) and registration with the Thai Clinical Trials Registry (TCTR20230816005) on 16 August 2023, the study was conducted in adherence to the Declaration of Helsinki and the International Council for Harmonization’s Good Clinical Practice (ICH-GCP) guidelines. We performed an investigator-blinded randomized controlled trial.

### Participants

Eligible patients were aged ≥ 18 years undergoing exploratory hysterectomy for benign or malignant conditions. They were recruited during September 2023 through February 2024, with informed consent being obtained from all participants. Exclusion criteria were allergy to MOM; intraoperative complications or comorbidities requiring prolonged restriction of oral intake; gastroesophageal reflux disease, peptic ulcer, chronic constipation, or bowel obstruction; and prior pelvic radiation.

### Sample size and power

For the primary endpoint—time from surgery to resumption of a solid diet—we based the sample size on Schilder et al. [14]. Women who were permitted early sipping resumed a regular diet at 1.88 ± 0.14 days, significantly sooner than controls [14]. We anticipated that the MOM group would resume solid diet at 1.55 days, or 0.33 days (8 h) earlier than controls, with a standard deviation of 0.564 days. With 90% power, α = 0.05, β = 0.1, and 20% attrition, the required sample was 160 women.

### Randomization and interventions

After surgery, eligible participants were randomized 1:1 in blocks of 4 into 2 groups by a sequence generated before study initiation, with allocation concealed and no participant interaction. The control group received standard ERAS, which included preoperative counseling, carbohydrate loading, avoidance of prolonged fasting, multimodal analgesia with opioid minimization, early mobilization, and early oral feeding: postoperative sips of liquids advancing to solids as tolerated in the absence of vomiting. The intervention group received ERAS plus MOM: 15 mL on postoperative day 0, then 30 mL twice daily until first bowel movement or passage of flatus, after which standard ERAS continued. A designated research nurse administered MOM.

### Outcomes and follow-up 

We collected demographic characteristics, operative details, and postoperative outcomes, which included bowel function, time from the end of surgery to resumption of a well-tolerated solid diet, and time to first flatus. We also recorded the use of additional bowel stimulants, which were permitted at the discretion of the treating physician and documented for analysis. In addition, eating and daily activity satisfaction scores, length of hospital stay, and MOM-related adverse events were assessed. Surgical complications were monitored for 30 days.

### Statistical analysis

Analyses used IBM SPSS Statistics version 29 (IBM Corp, Armonk, NY, USA) under an intention-to-treat framework. Results were summarized as counts (percentages) and continuous variables were presented as mean ± standard deviation (SD) or median (interquartile range), as appropriate. Normality was assessed using histogram inspection and the Kolmogorov–Smirnov test. Continuous variables were compared with Student’s *t* test or the Mann–Whitney *U* test, and categorical variables with the chi-square or Fisher’s exact test. A *P* value < 0.05 indicated statistical significance.

### Operational definitions

#### POI diagnosis

POI was diagnosed using Vather criteria [15], which require at least 2 of 5 findings:

1. nausea with or without vomiting;
2. inability to tolerate a solid or semi-liquid diet for 24 h or longer;
3. no flatus or defecation for 24 h or more;
4. abdominal distention;
5. radiological evidence of ileus.

#### Well-tolerated solid diet

Defined as the ability to consume at least 50% of a solid meal without vomiting.

#### Discharge criteria

Defined as independent ambulation, tolerance of a solid diet, and pain control with oral analgesics.

#### Length of stay

Time from the end of surgery until all discharge criteria were met.

#### Patient-reported outcomes

Eating satisfaction and daily activity satisfaction were assessed using a visual analog scale ranging from 0 to 10, with higher scores indicating greater satisfaction and better perceived recovery.

#### Severe adverse events related to MOM

Severity was defined using prespecified clinically relevant criteria, including severe diarrhea (> 6 episodes/day), dehydration (urine output < 0.5 mL/kg/hour), hypotension (blood pressure < 90/60 mmHg), and symptoms suggestive of hypermagnesemia, such as muscle weakness, arrhythmia, and respiratory depression (respiratory rate < 12 breaths per minute).

## Results

### Participant flow and baseline characteristics

**Fig. 1:**
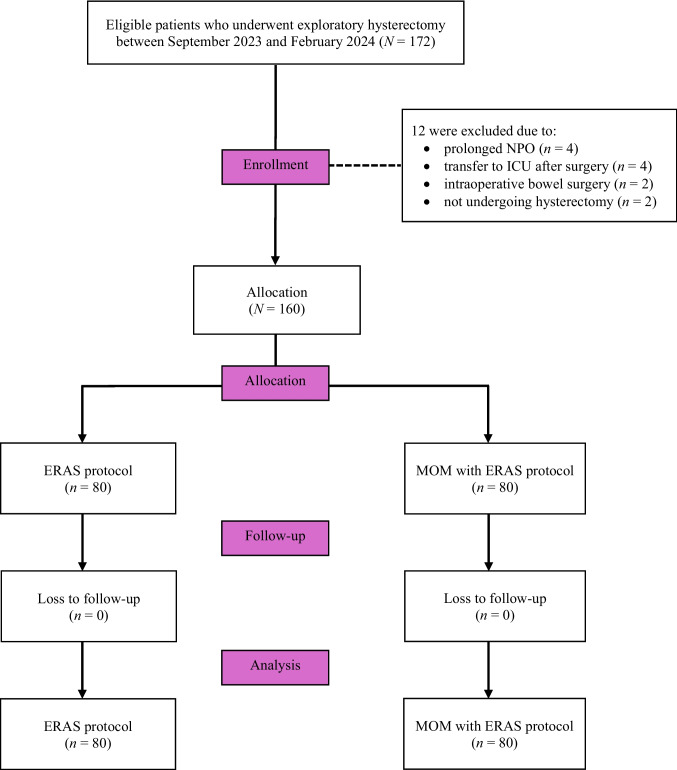
Participant flow diagram for the randomized trial, September 2023–February 2024. Abbreviations: *ERAS* enhanced recovery after surgery, *ICU* intensive care unit, *MOM* milk of magnesia, *NPO* nothing by mouth

Of 172 eligible patients, 12 were excluded, as shown in Fig. 1. One hundred sixty participants were randomized equally to 2 arms (*n* = 80 per arm). Baseline characteristics, surgical methods, and perioperative medications are summarized in Table 1. The majority had benign gynecologic conditions (62.5%; *n* = 100). Malignancies comprised 37.5% (*n* = 60): endometrial cancer (*n* = 34), ovarian cancer (*n* = 16), cervical cancer (*n* = 8), and leiomyosarcoma (*n* = 2). There were no between-group differences in preoperative care, including use of analgesics, proton pump inhibitors, antiemetics, bowel preparation, or fasting duration. Similarly, intraoperative drug use, anesthetic methods, and postoperative antiemetic or opioid administration did not differ. The groups were comparable in surgical approach (midline or transverse incision), operative time, and intraoperative blood loss; no participant developed hypothermia.

**Table 1:** Baseline characteristics, surgical methods, and perioperative medications by randomized group (standard ERAS vs ERAS with MOM)

Variables	Control group *n* = 80	MOM group *n* = 80	*P* value
Age, y	51.61 ± 12.151	51.44 ± 10.520	0.923
BMI, kg/m^2^	23.365 ± 4.392	25.405 ± 5.931	0.014
Diseases			
Benign	50 (62.5)	50 (62.5)	1.000
Malignant	30 (37.5)	30 (37.5)	
ASA classification			
I	26 (32.5)	24 (30.0)	0.515
II	48 (60.0)	53 (66.3)	
III	6 (7.5)	3 (3.8)	
IV	0	0	
Comorbidities			
Diabetes mellitus, *n* = 18	10 (12.5)	8 (10.0)	0.617
Hypertension, *n* = 44	20 (25.0)	24 (30.0)	0.497
Dyslipidemia, *n* = 40	18 (22.5)	22 (27.5)	0.465
Stroke, *n* = 6	6 (7.5)	0	0.028
Alcohol, current user	0	7 (8.8)	0.026
Smoking, current user	0	1 (1.3)	0.316
Preoperative hemoglobin, g/dL	11.898 ± 1.535	12.109 ± 1.501	0.674
Preoperative painkillers within 2 wk	6 (7.5)	5 (6.3)	0.755
Preoperative proton pump inhibitor	29 (36.3)	28 (35.0)	0.869
Preoperative antiemetic	23 (28.7)	20 (25.0)	0.593
Preoperative bowel preparation	10 (12.5)	8 (10.0)	0.631
Fasting time (h)			
From regular diet	17.422 ± 1.680	17.577 ± 1.995	0.595
From sip water	4.268 ± 1.4939	4.502 ± 1.6358	0.345
Incision type			0.990
Midline	37 (46.3)	38 (47.5)	
Transverse	43 (53.8)	42 (52.6)	
Adhesiolysis	17 (21.3)	18 (22.5)	0.848
Surgical drain	2 (2.5)	0	0.155
Surgical time, min	143.65 ± 54.828	144.73 ± 49.112	0.896
Estimated blood loss, mL	276.19 ± 276.503	329.88 ± 314.270	0.253
General anesthesia component	37 (46.3)	38 (47.5)	0.732
Intravenous fluid positive, mL	2160.05 ± 976.23	2221.41 ± 1045.61	0.702
Intraoperative paracetamol	5 (6.3)	8 (10.0)	0.385
Intraoperative morphine	9 (11.3)	10 (12.5)	0.807
Intraoperative morphine, mg	5.39 ± 2.826	5.5 ± 3.300	0.938
Intraoperative fentanyl	71 (88.8)	66 (82.5)	0.260
Intraoperative fentanyl, mcg	69.01 ± 32.034	65.08 ± 28.494	0.450
Intraoperative xylocaine	5 (6.3)	6 (7.5)	0.755
Intraoperative ondansetron	58 (72.5)	51 (63.7)	0.235
Intraoperative ondansetron, mg	7.9 ± 0.7888	7.88 ± 0.621	0.918
Intraoperative metoclopramide	5 (6.3)	8 (10.0)	0.385
Postoperative opioids	8 (10.0)	11 (13.8)	0.463
Postoperative antiemetics	42 (52.5)	39 (48.8)	0.635
Postoperative simethicone	47 (58.8)	47 (58.8)	0.603
Routine per physician preference	46	47	
On patient request	1	0	
Routine postoperative omeprazole	15 (18.8)	16 (20.0)	0.841

Unless otherwise indicated, data are mean ± SD or *n* (%). *P* values compare groups. Student’s *t* test or the Mann–Whitney *U* test was used for continuous variables, and the chi‑square or Fisher’s exact test for categorical variables. Preoperative analgesic use was assessed within 2 weeks before surgery. Fasting time reflects hours since last regular diet and last sip of water. “General anesthesia component” indicates use of general anesthesia within a multimodal anesthetic technique. “Intravenous fluid positive” denotes intraoperative intravenous fluid volume administered

*ASA* American society of anesthesiologists physical status classification system, *BMI* body mass index, *ERAS* enhanced recovery after surgery, *MOM* milk of magnesia, *SD* standard deviation

### Postoperative outcomes

As shown in Table 2, continuous variables are presented as mean ± SD. The primary outcome, time to well-tolerated solid diet, was significantly shorter in the MOM group compared with the control group (20.6 ± 10.9 vs 28.8 ± 16.3 h; *P* < 0.001). The incidence of POI was significantly lower in the MOM group (3.8% vs 27.5%; *P* < 0.001), and time to first flatus was also shorter (17.1 ± 7.9 vs 22.3 ± 10.8 h; *P* < 0.001).

**Table 2:** Postoperative outcomes by randomized group (standard ERAS vs ERAS with MOM)

Variables	Control group *n* = 80	MOM group *n* = 80	*P* value
Postoperative nausea	44 (55.0)	30 (37.5)	0.026
Emesis	30 (37.5)	14 (17.5)	0.005
Bloating	30 (37.5)	14 (17.5)	0.005
POI occurrence	22 (27.5)	3 (3.8)	< 0.001
Upper	20	2	
Lower	2	1	
Time to first flatus (h)	22.309 ± 10.8827	17.101 ± 7.9619	0.001
Time to first belch (h)	15.391 ± 10.9448	12.482 ± 7.565	0.051
Additional bowel stimulants	53 (66.3)	0	< 0.001
Time to first soft diet (h)	12.001 ± 6.698	13.093 ± 6.444	0.295
Time to well‑tolerated solid diet (h)	28.800 ± 16.3435	20.691 ± 10.9635	< 0.001
Time to off IV fluids (h)	18.104 ± 4.914	17.765 ± 5.231	0.673
Time to off Foley catheter (h)	25.969 ± 20.967	23.826 ± 16.891	0.480
Pain score, maximum level of the day			
POD 0	2.9 ± 2.004	3.13 ± 1.885	0.466
POD 1	1.91 ± 1.314	2.24 ± 1.204	0.105
Eating satisfaction score	7.81 ± 1.342	8.71 ± 0.860	< 0.001
Daily activities satisfaction score	8.35 ± 1.02	8.98 ± 0.811	< 0.001
Length of hospital stay (h)	81.602 ± 49.837	72.71 ± 22.765	0.149

Unless otherwise indicated, data are mean ± SD or *n* (%). Times are measured in hours from the end of surgery. “Upper” and “Lower” denote the reported location of POI. Time to well-tolerated solid diet was defined as the time from the end of surgery to the ability to consume at least 50% of a solid meal without vomiting. Group comparisons used Student’s *t* test or the Mann–Whitney *U* test for continuous variables, and the chi-square or Fisher’s exact test for categorical variables. A *P* value < 0.05 was considered statistically significant

*ERAS* enhanced recovery after surgery, *IV* intravenous, *MOM* milk of magnesia, *POD* postoperative day, *POI* postoperative ileus, *SD* standard deviation

Postoperative nausea (37.5% vs 55.0%; *P* = 0.026), emesis (17.5% vs 37.5%; *P* = 0.005), and bloating (17.5% vs 37.5%; *P* = 0.005) were each significantly less frequent in the MOM group than in the control group (Table 2). No participants in the MOM group required additional bowel stimulants, whereas 66.3% of those in the control group did. Length of hospital stay was shorter in the MOM group (72.7 ± 22.7 vs 81.6 ± 49.8 h), although this difference was not statistically significant (*P* = 0.149). 

Patient-reported outcomes favored the MOM group, with significantly higher eating satisfaction (8.7 ± 0.8 vs 7.8 ± 1.3; *P* < 0.001) and daily activity satisfaction scores (8.9 ± 0.8 vs 8.3 ± 1.0; *P* < 0.001).

### Safety

The incidences of specific postoperative adverse events, including nausea, emesis, bloating, and postoperative ileus, are summarized in Table 2. Overall, MOM-related adverse events occurred in 8 of 80 patients (10.0%) in the MOM group and were all mild. No severe MOM-related adverse events occurred: no patient experienced severe diarrhea (> 6 episodes/day), dehydration, hypotension, or symptoms suggestive of hypermagnesemia. The 8 affected patients experienced increased bowel frequency (approximately 3–4 bowel movements per day); these episodes were not associated with large-volume diarrhea, did not impair daily activities, did not require additional treatment, and resolved after discontinuation of MOM. No major surgical complications occurred within 30 days in either group. No participant withdrew from the study because of MOM-related adverse events.

## Discussion

Adding MOM to standard ERAS was associated with a significant reduction in POI and faster recovery of bowel function, as reflected by earlier flatus, improved tolerance of solid diet, and fewer gastrointestinal symptoms, including nausea, vomiting, and abdominal distention. Notably, none of the patients in the MOM group required additional bowel stimulants, whereas a substantial proportion of patients in the control group did. Although the use of additional bowel stimulants in the control group may have acted as a confounding factor, the consistently superior outcomes in the MOM group suggest that the observed benefits were unlikely to be solely attributable to rescue bowel stimulation. These findings may be explained by the pharmacological effects of MOM. As an osmotic laxative, magnesium hydroxide increases water retention within the intestinal lumen, thereby stimulating peristalsis and facilitating bowel movement [16], which may promote earlier gastrointestinal recovery and improved tolerance of solid diet. In addition, similar bowel stimulation strategies, including osmotic laxatives and early feeding within ERAS protocols, have been shown to enhance postoperative gastrointestinal recovery [11, 12]. Furthermore, MOM may exert dual effects during the postoperative period: on postoperative day 0, a lower dose may function as an antacid, reducing gastric acidity and alleviating early postoperative bloating, whereas from postoperative day 1 onward, higher doses act predominantly as a laxative, promoting bowel activity and facilitating gastrointestinal recovery.

Our findings align with Fanning et al., who studied 707 major gynecologic operations and administered 30 mL MOM twice daily until bowel movement occurred [2]. POI occurred in less than 1% of patients (6 of 707) [2], which is lower than the 3.8% observed in the present trial. Differences likely reflect surgical mix and invasiveness. In Fanning et al., approximately 87% of procedures were laparoscopic, and 29.4% were adnexal surgeries, both of which generally have shorter operative times and less bowel manipulation than hysterectomy [2]. These factors likely reduced bowel irritation and explain their lower POI rate compared with our cohort.

Schilder et al. reported that women undergoing major abdominal surgery began a clear liquid diet on postoperative day 1 and advanced to a regular diet after tolerating 500 mL of clear liquids [14]. This early feeding reduced time to solid diet consumption from 2.72 ± 0.14 days to 1.88 ± 0.14 days (*P* < 0.001) [14]. In our trial, time to solid diet tolerance occurred at 20.6 ± 10.9 h with MOM versus 28.8 ± 16.3 h with standard ERAS, with both earlier than in Schilder et al. We attribute this acceleration to standard ERAS elements, including preoperative carbohydrate loading, allowance of clear liquids until 2 h before surgery, early ambulation, optimal pain control, multimodal analgesia, and reduced opioid exposure [11, 12]. Together, these components likely enabled both groups to tolerate solids sooner than in prior reports.

Time to first flatus was similarly accelerated in our cohort: 17.1 ± 7.9 h in the MOM group, which is faster than previous reports. For comparison, a radical hysterectomy series administered 30 mL MOM twice daily starting on postoperative day 1, with a bisacodyl suppository on postoperative day 2 [13]. The median time to flatus was 3 days (range 2–3 days) [13]. These data demonstrate that integrating MOM into ERAS pathways expedites bowel function return, enabling earlier flatus and solid diet tolerance. 

Although the absolute differences in time to first flatus and tolerance of solid diet were measured in hours rather than days, these improvements may still be clinically meaningful within an ERAS pathway. Earlier recovery of gastrointestinal function may improve patient comfort, facilitate oral intake, reduce gastrointestinal symptoms, and decrease the need for additional bowel stimulants. Moreover, the substantially lower incidence of postoperative ileus in the MOM group suggests that these benefits extend beyond statistical significance and may translate into meaningful clinical improvement.

Length of hospital stay was shorter with MOM (72.7 ± 22.7 h) than with standard ERAS (81.6 ± 49.8 h), but not significantly so. Our institution routinely discharges hysterectomy patients on postoperative day 3, even when they tolerate solids or pass flatus by day 1 or 2. This practice likely masked between-group differences in length of stay despite earlier bowel recovery with MOM. By contrast, other studies have shown significant length of stay reductions with MOM [13, 17].

Eating satisfaction was significantly higher with MOM (8.7 ± 0.8 vs 7.8 ± 1.3; *P* < 0.001), as was daily activities satisfaction (8.9 ± 0.8 vs 8.3 ± 1.0; *P* < 0.001). These improvements likely reflect reduced nausea, vomiting, and bloating, plus earlier bowel function return. Earlier flatus and bowel movements facilitated faster solid diet tolerance, improving satisfaction with both eating and daily activities.

This study has several strengths. It is a prospective randomized controlled trial of abdominal hysterectomy for benign and malignant conditions, with largely comparable baseline characteristics between groups. MOM is cost-effective and covered by all healthcare plans, enabling universal access without financial burden. Outcomes were clearly defined, including POI, well-tolerated solid diet, discharge criteria, and length of stay. However, certain outcomes were subjective, particularly time to first flatus. Patients may pass flatus during sleep or other unobserved activities, so recorded times may exceed actual times, potentially biasing recovery-time assessments. Additionally, the lack of participant blinding may have introduced bias in patient-reported outcomes, such as satisfaction scores, as participants were aware of their treatment allocation. Furthermore, this was a single-center study, which may limit the generalizability of the findings. In addition, some baseline characteristics, including BMI, history of stroke, and current alcohol use, differed between groups. Although these differences were unlikely to fully explain the observed bowel recovery outcomes, they may represent potential confounders. Finally, adverse events were not graded using a standardized framework such as CTCAE. Instead, safety assessment focused on clinically relevant MOM-related gastrointestinal adverse events, which may limit direct comparison with studies using formal adverse-event grading systems. These limitations should be considered when interpreting the findings.

## Conclusion

Adding MOM to ERAS was associated with reduced POI incidence and faster recovery of bowel function in patients undergoing abdominal hysterectomy for benign or malignant conditions.

## Data Availability

Correspondence and requests for materials should be addressed to WK.

## References

[1] Venara A, Neunlist M, Slim K, Barbieux J, Colas PA, Hamy A et al (2016) Postoperative ileus: pathophysiology, incidence, and prevention. J Visc Surg 153(6):439–446 27666979 10.1016/j.jviscsurg.2016.08.010

[2] Fanning J, Hojat R (2011) Safety and efficacy of immediate postoperative feeding and bowel stimulation to prevent ileus after major gynecologic surgical procedures. J Am Osteopath Assoc 111(8):469–472 21862754 10.7556/jaoa.2011.111.8.469

[3] Güngördük K, Özdemir İA, Güngördük Ö, Gülseren V, Gokçü M, Sancı M (2017) Effects of coffee consumption on gut recovery after surgery of gynecological cancer patients: a randomized controlled trial. Am J Obstet Gynecol 216(2):145.e1-145.e7 27780709 10.1016/j.ajog.2016.10.019

[4] Li Z-L, Zhao B-C, Deng W-T, Zhuang P-P, Liu W-F, Li C et al (2020) Incidence and risk factors of postoperative ileus after hysterectomy for benign indications. Int J Colorectal Dis 35(11):2105–2112 32699935 10.1007/s00384-020-03698-5

[5] Eamudomkarn N, Kietpeerakool C, Kaewrudee S, Jampathong N, Ngamjarus C (2018) Lumbiganon P (2018) Effect of postoperative coffee consumption on gastrointestinal function after abdominal surgery: a systematic review and meta-analysis of randomized controlled trials. Sci Rep 8(1):17349 30478433 10.1038/s41598-018-35752-2 PMC6255780

[6] Jirawongprapa Y, Chotboon C, Songthamwat S, Summart U, Songthamwat M (2023) Effects of caffeine dose on bowel function recovery following gynecologic cancer surgery: a randomized double-blind controlled trial. Thai J Obstet Gynaecol 31(4):265–272

[7] Kadirogullari P, Seckin KD, Yalcin Bahat P, Aytufan Z (2022) The effect of chewing gum on bowel function postoperatively in patients with total laparoscopic hysterectomy: a randomised controlled trial. J Obstet Gynaecol 42(5):1192–1197 34379539 10.1080/01443615.2021.1941821

[8] Sillapasa C, Jeerasap R, Tangsiriwatthana T (2023) Metoclopramide for Preventing Ileus after Benign Gynecologic Surgery: A randomized controlled trial. Thai Journal of Obstetrics and Gynaecology 31(3):192–200

[9] Chaiyakunapruk N, Kitikannakorn N, Nathisuwan S, Leeprakobboon K, Leelasettagool C (2006) The efficacy of ginger for the prevention of postoperative nausea and vomiting: a meta-analysis. Am J Obstet Gynecol 194(1):95–99 16389016 10.1016/j.ajog.2005.06.046

[10] Pongsupanimit P, Chaikomin R, Tripatara P, Achariyapota V, Viriyapak B, Kanpetpanao S et al (2025) The impact of ginger on preventing postoperative ileus after hysterectomy under the enhanced recovery after surgery protocol: a randomized controlled trial. Thai J Obstet Gynaecol 33(1):53–63

[11] Nelson G, Bakkum-Gamez J, Kalogera E, Glaser G, Altman A, Meyer LA et al (2019) Guidelines for perioperative care in gynecologic/oncology: enhanced recovery after surgery (ERAS) society recommendations-2019 update. Int J Gynecol Cancer 29(4):651–668 30877144 10.1136/ijgc-2019-000356

[12] Gómez-Hidalgo NR, Pletnev A, Razumova Z, Bizzarri N, Selcuk I, Theofanakis C et al (2023) European enhanced recovery after surgery (ERAS) gynecologic oncology survey: status of ERAS protocol implementation across Europe. Int J Gynaecol Obstet 160(1):306–312 35929452 10.1002/ijgo.14386

[13] Fanning J, Yu-Brekke S (1999) Prospective trial of aggressive postoperative bowel stimulation following radical hysterectomy. Gynecol Oncol 73(3):412–414 10366469 10.1006/gyno.1999.5401

[14] Schilder JM, Hurteau JA, Look KY, Moore DH, Raff G, Stehman FB et al (1997) A prospective controlled trial of early postoperative oral intake following major abdominal gynecologic surgery. Gynecol Oncol 67(3):235–240 9441769 10.1006/gyno.1997.4860

[15] Vather R, Trivedi S, Bissett I (2013) Defining postoperative ileus: results of a systematic review and global survey. J Gastrointest Surg 17(5):962–972 23377782 10.1007/s11605-013-2148-y

[16] Brunton LL, Hilal-Dandan R, Knollmann BC (eds) (2018) Goodman & Gilman’s the pharmacological basis of therapeutics, 13th ed. McGraw-Hill Education, New York

[17] Tatar C, Hinckley S, Holubar SD, Líška D, Delaney CP, Steele SR et al (2022) Does milk of magnesia impact length of hospital stay after major colorectal resection. ANZ J Surg 93 10.1111/ans.18196 36495072

